# Supplementary material for: Spectral study of COVID-19 pandemic in Japan: The dependence of spectral gradient on the population size of the community
Source: PLoS One. 2025 Jan 13;20(1):e0314233. doi: 10.1371/journal.pone.0314233 (PMC11730377; doi:10.1371/journal.pone.0314233)
Supplement: S1 Appendix — (DOCX) [file pone.0314233.s002.docx]

**S1 Appendix. Assignment of fundamental modes**

In general, the power spectrum is composed of a set of peaks, located at fundamental frequencies, *f*_I_, *f*_II_, …, *f*_R_, and their harmonics *m*_I_*f*_I_, *m*_II_*f*_II_, *m*_R_*f*_R_ (*m*_I_, *m*_II_, …, *m*_R_: positive integers), and contains all components at frequencies of the combinations $\left|m_{\text{I}}f_{\text{I}}+m_{\text{II}}f_{\text{II}}+\cdots+m_{\text{R}}f_{\text{R}}\right|$. Therefore, we attempted to assign the frequencies of the spectral lines shown in Fig 6 by using the relation $f=\left|m_{\text{I}}f_{\text{I}}+m_{\text{II}}f_{\text{II}}+m_{\text{III}}f_{\text{III}}\cdots\right|$. The results of these assignments are shown in Table 1.
