# Supplementary figures and images for: Spectral study of COVID-19 pandemic in Japan: The dependence of spectral gradient on the population size of the community
Source: PLoS One. 2025 Jan 13;20(1):e0314233. doi: 10.1371/journal.pone.0314233 (PMC11730377; doi:10.1371/journal.pone.0314233)

| 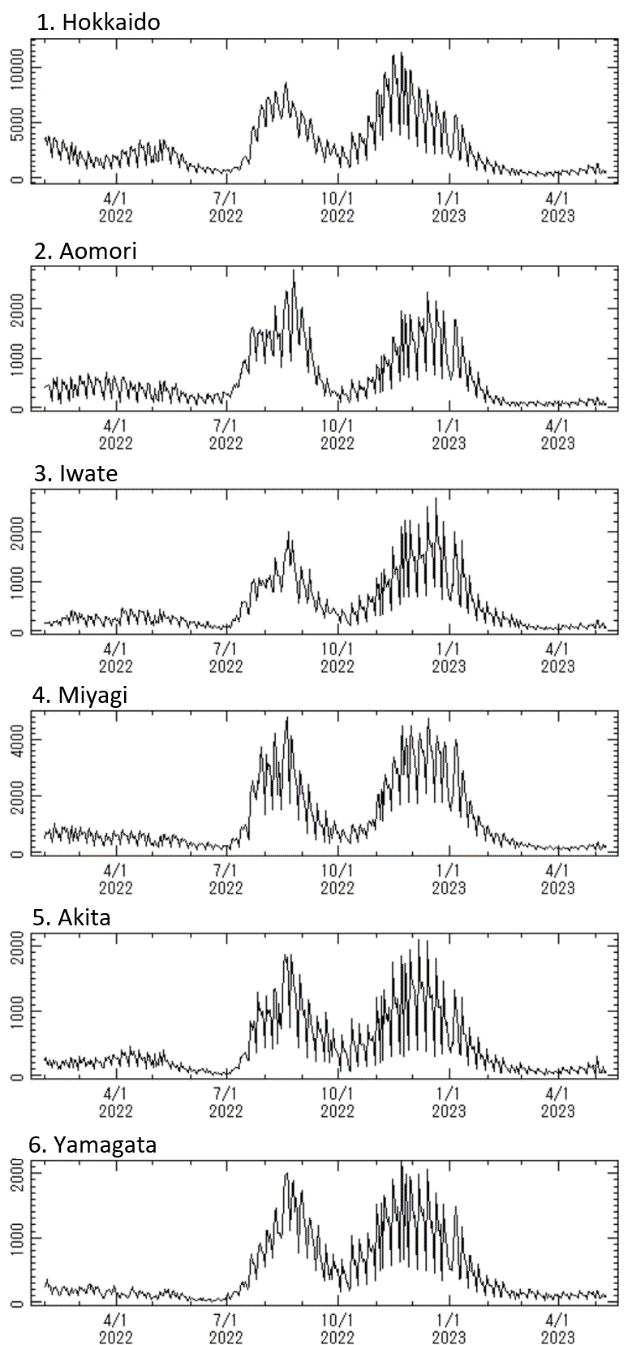 | 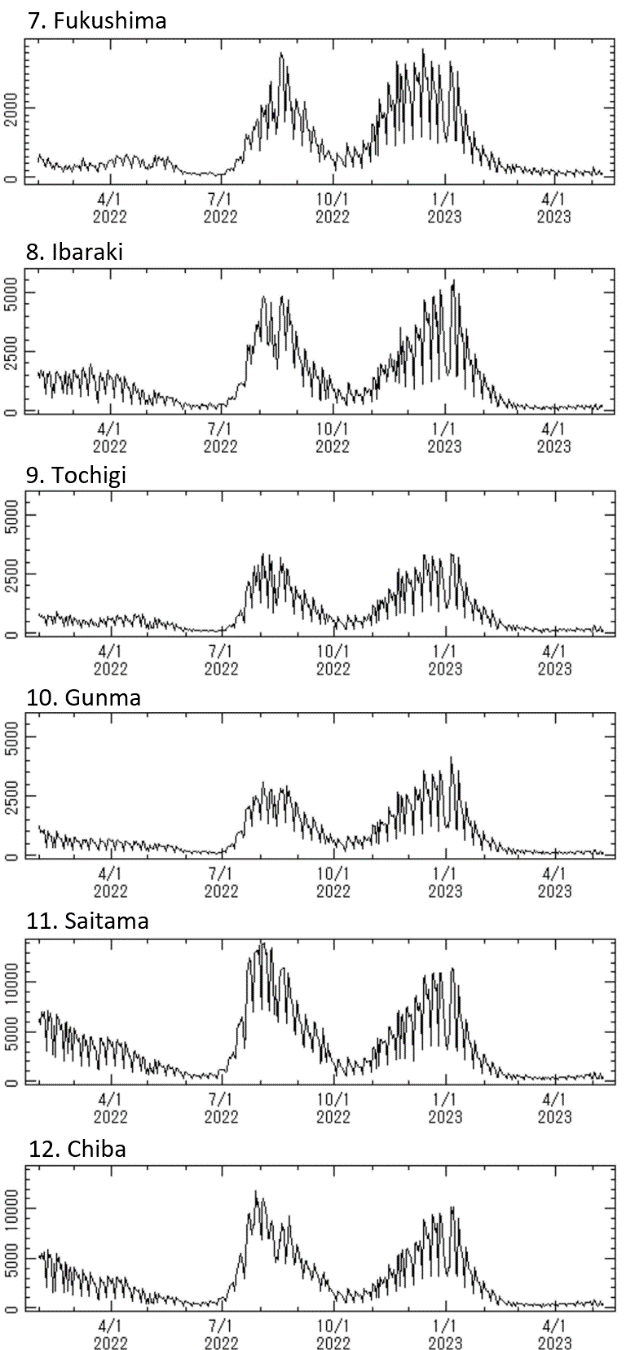 |
| --- | --- |

| 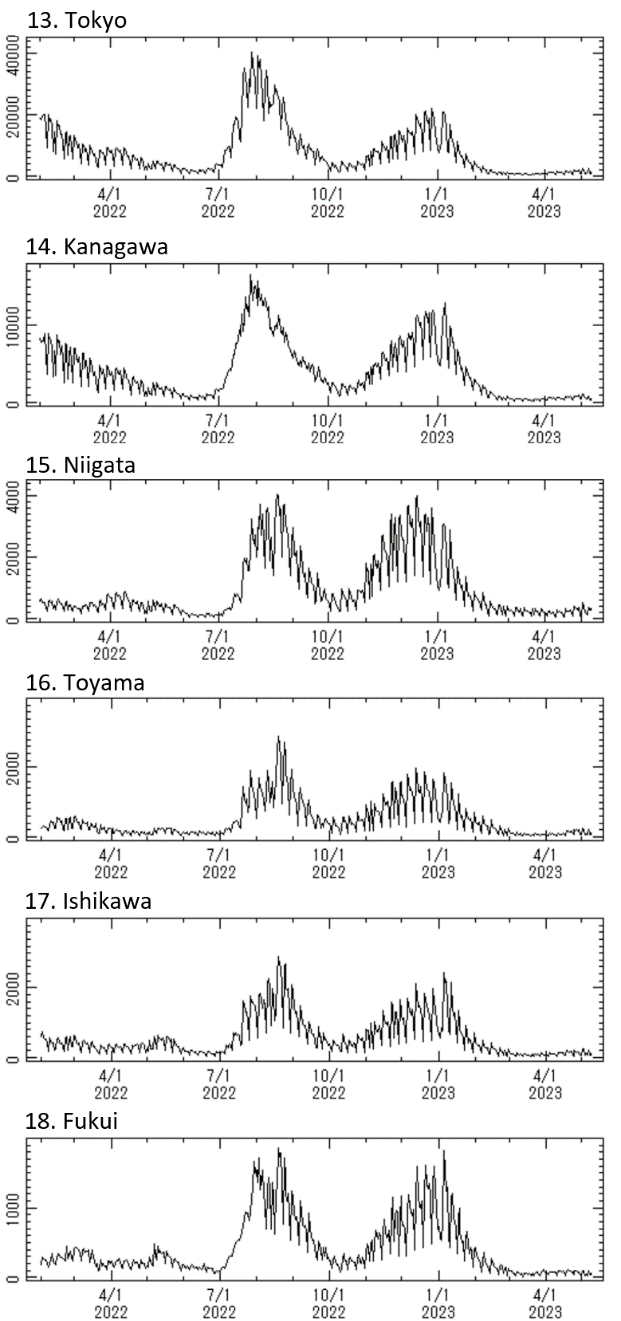 | 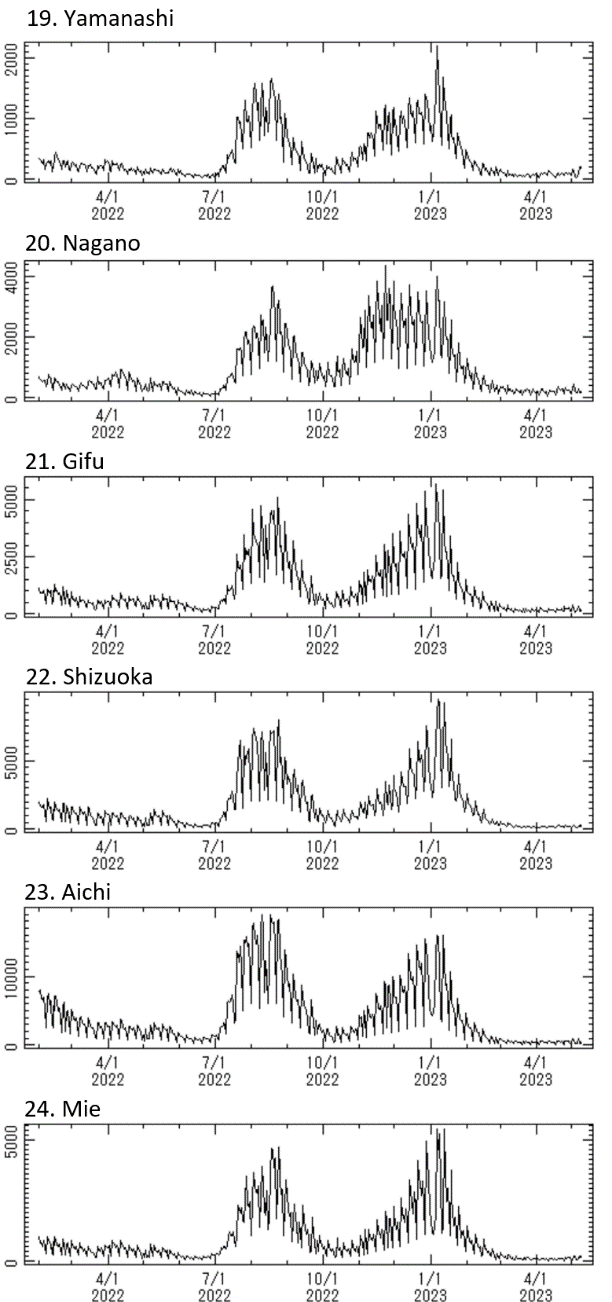 |
| --- | --- |

| 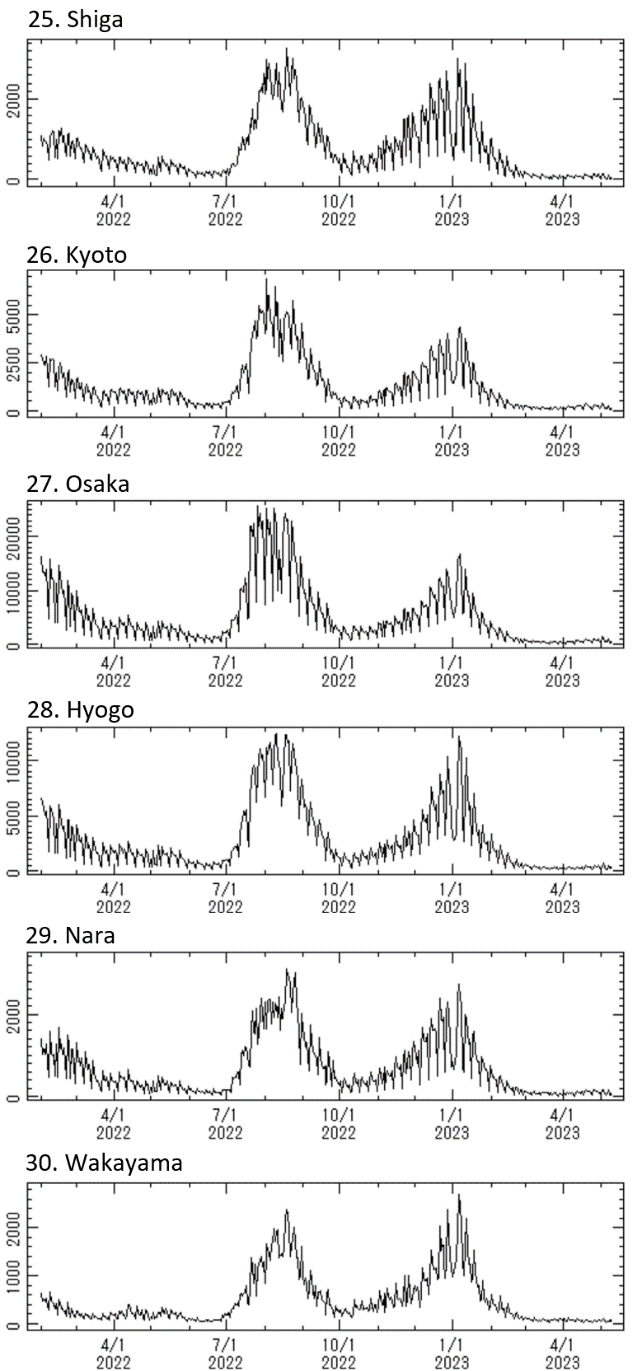 | 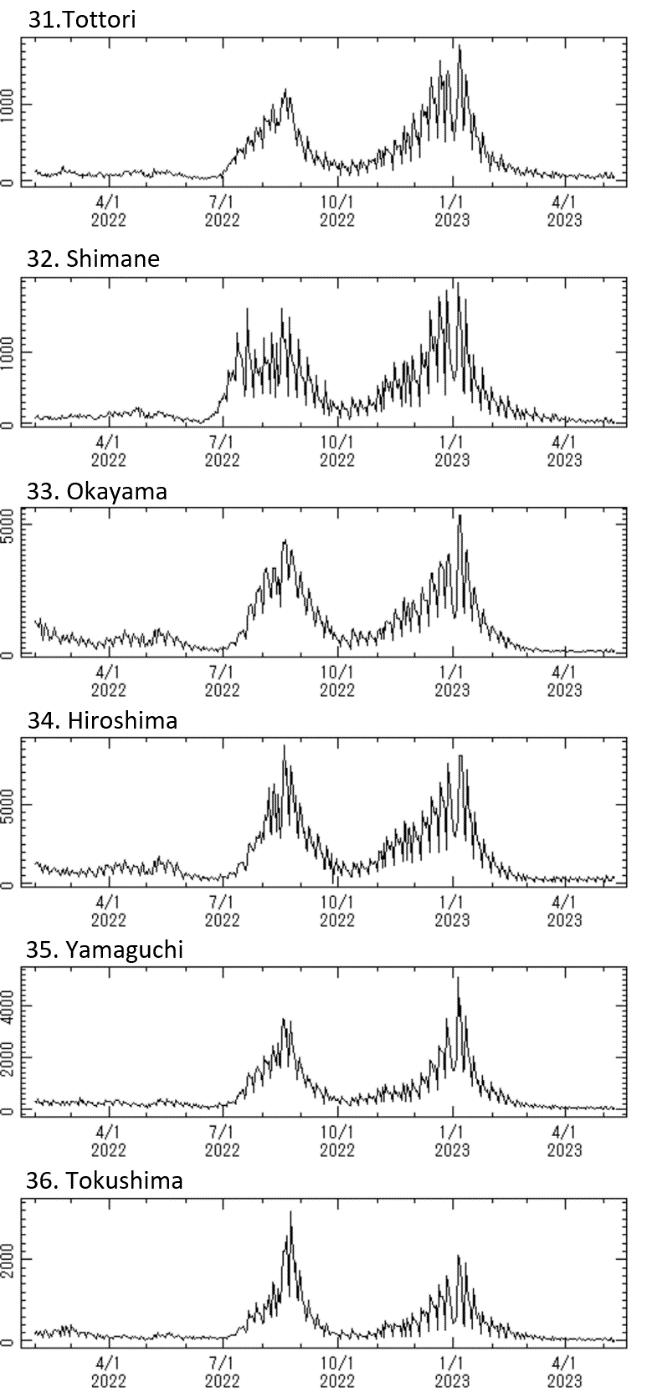 |
| --- | --- |

| 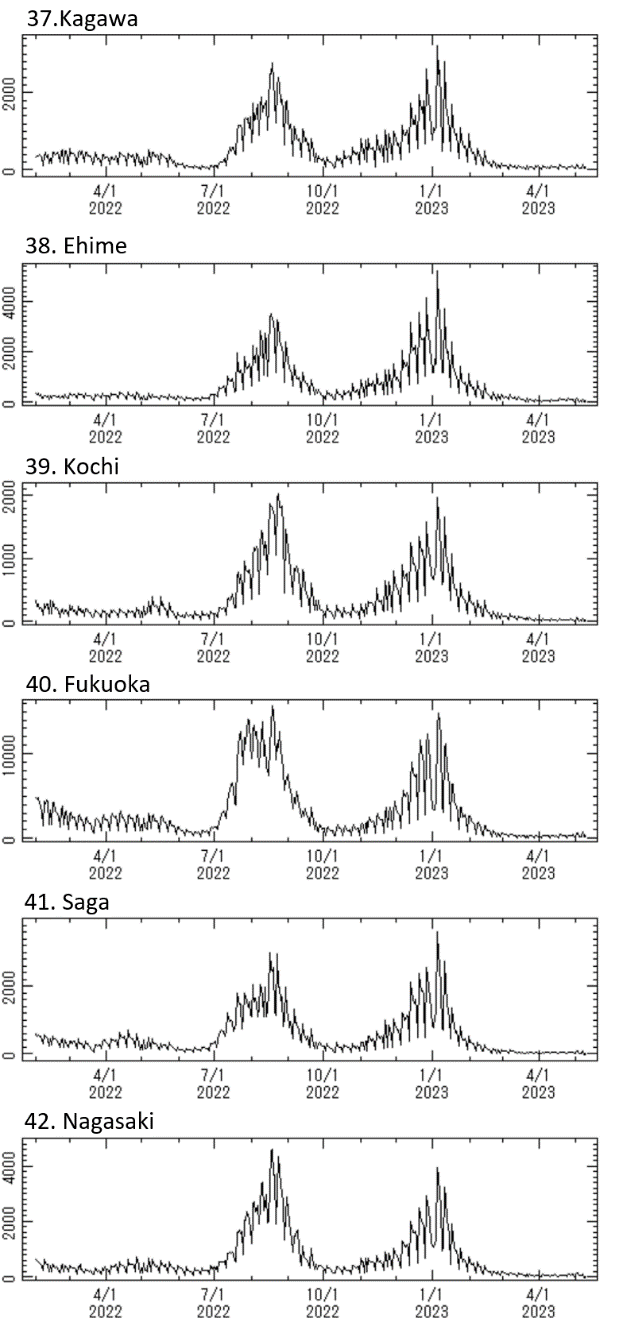 | 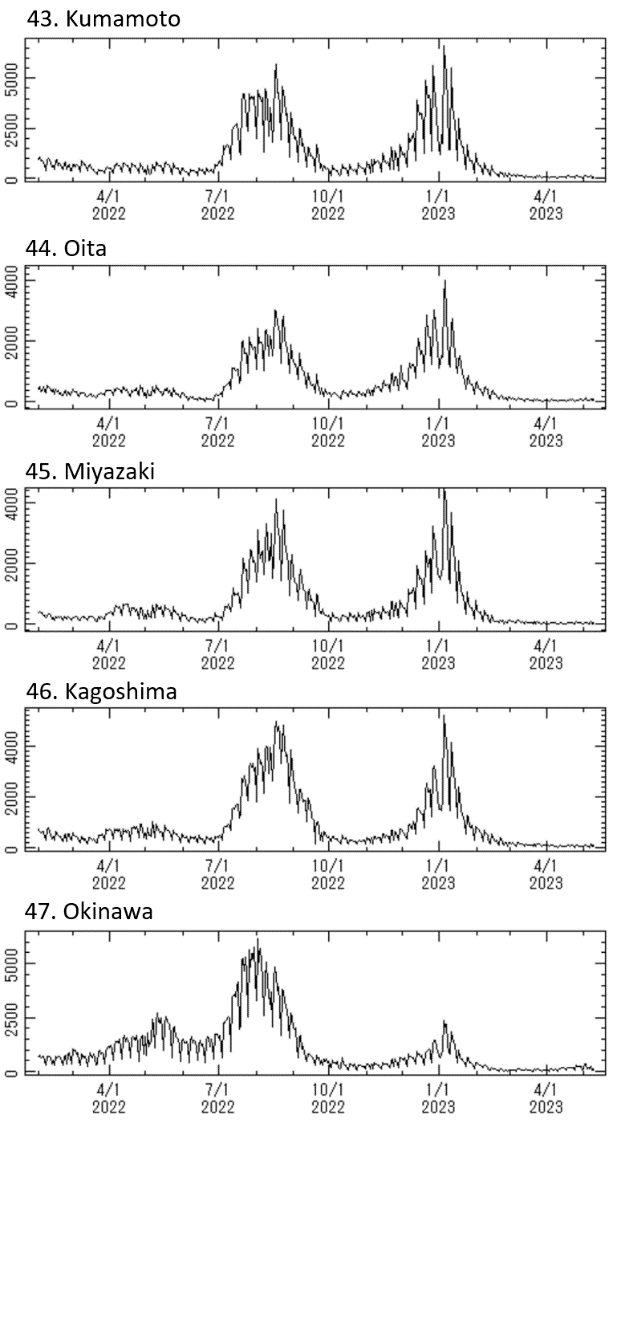 |
| --- | --- |

Supplement: S1 Fig — (DOCX) [file pone.0314233.s003.docx]

| 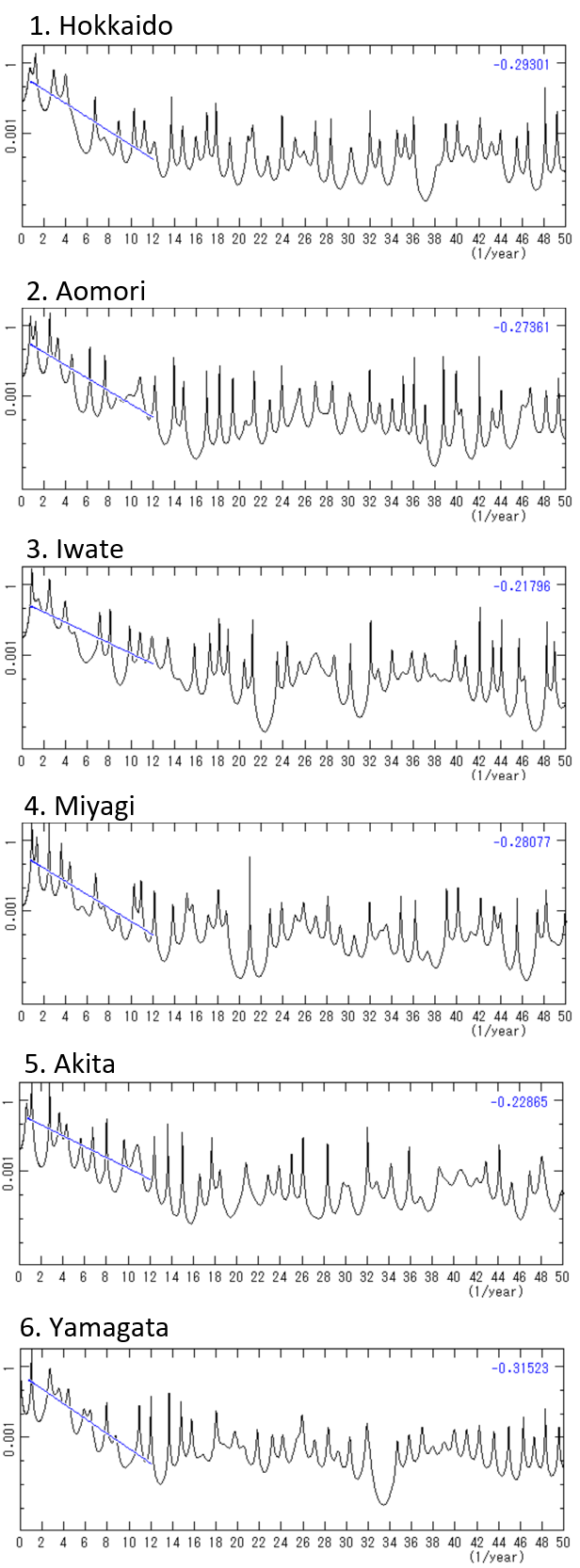 | 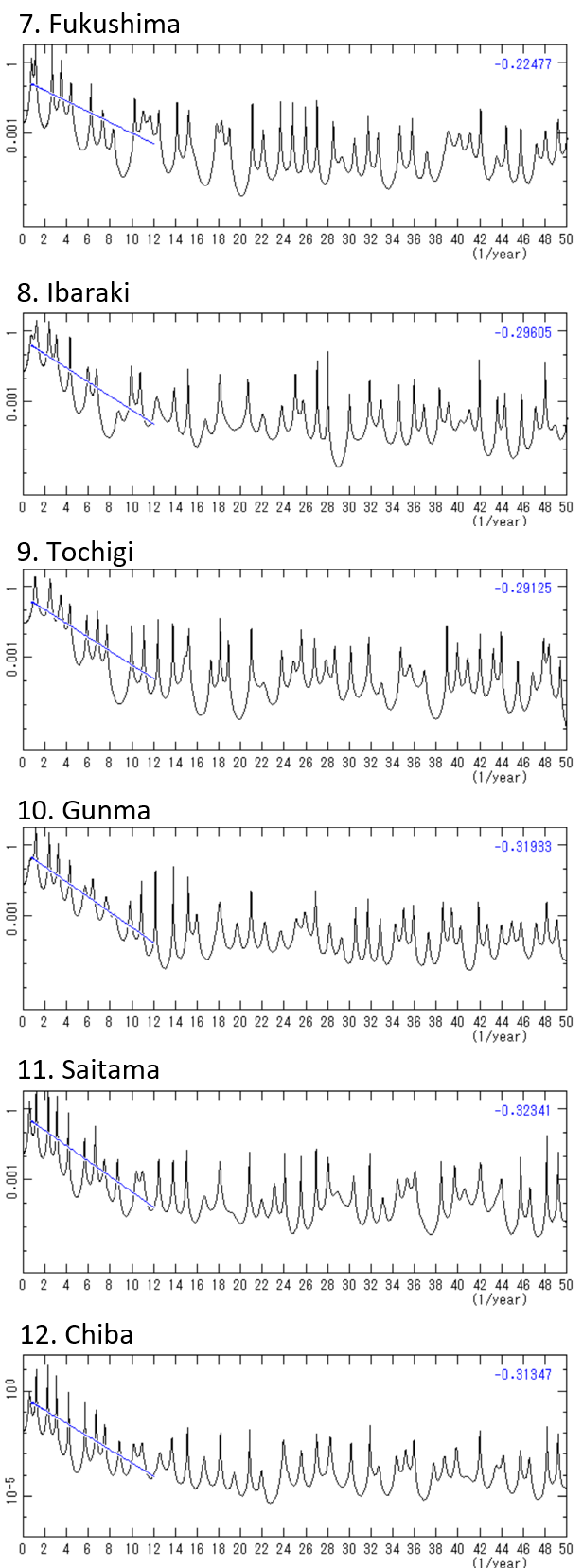 |
| --- | --- |

| 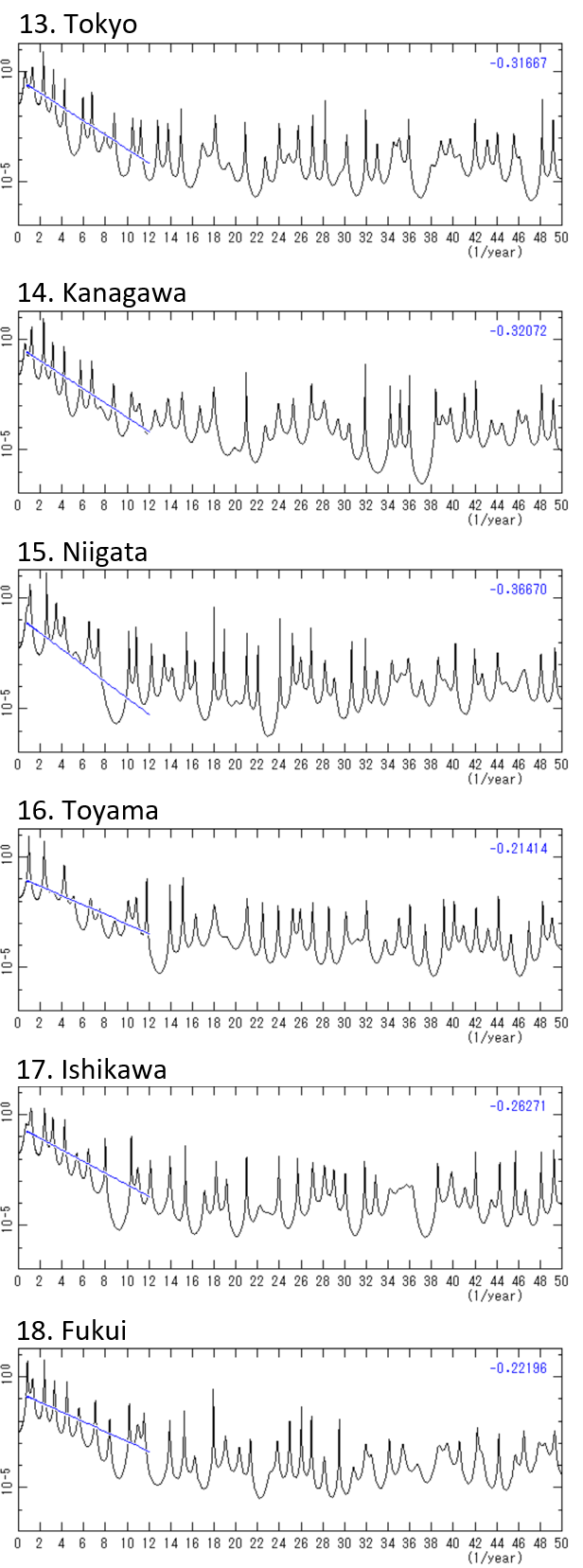 | 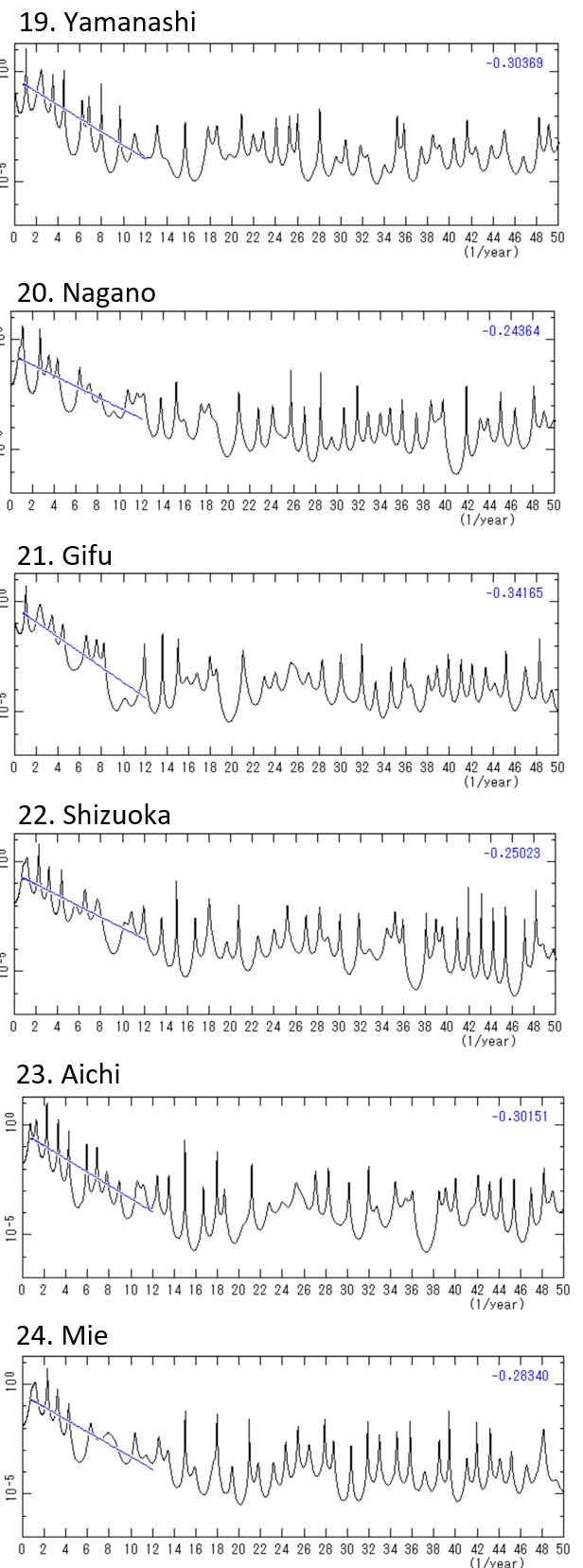 |
| --- | --- |

| 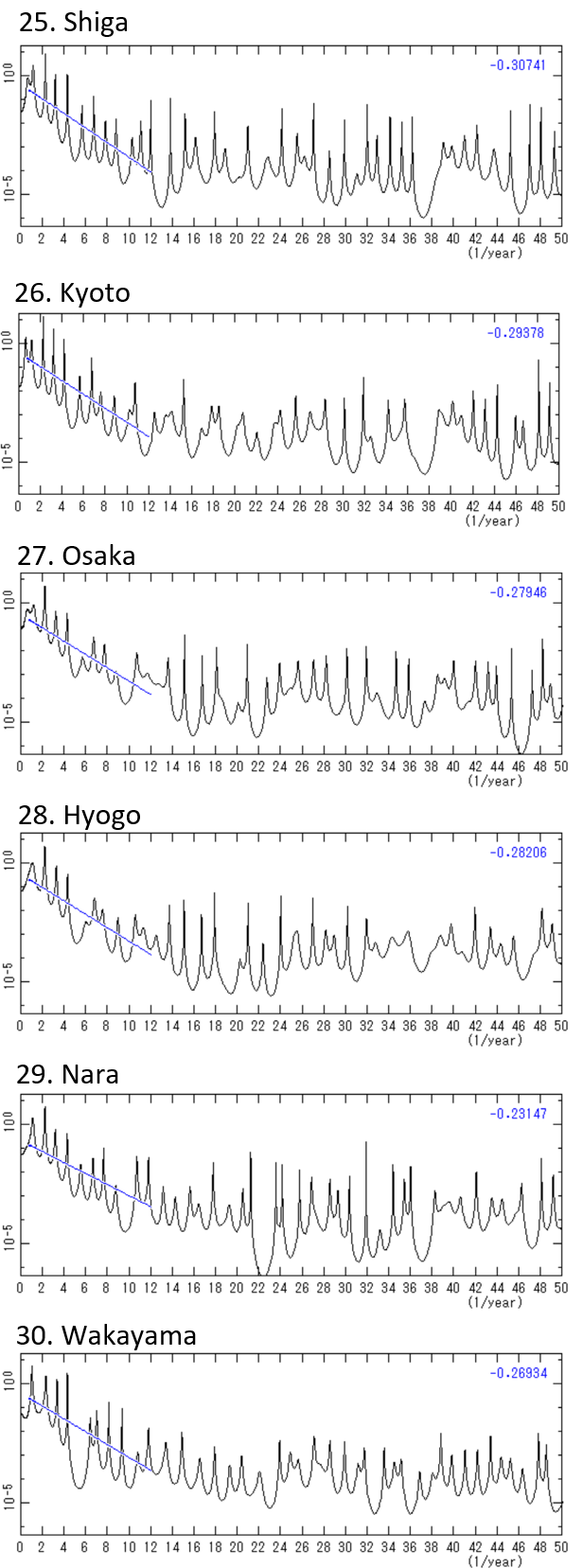 | 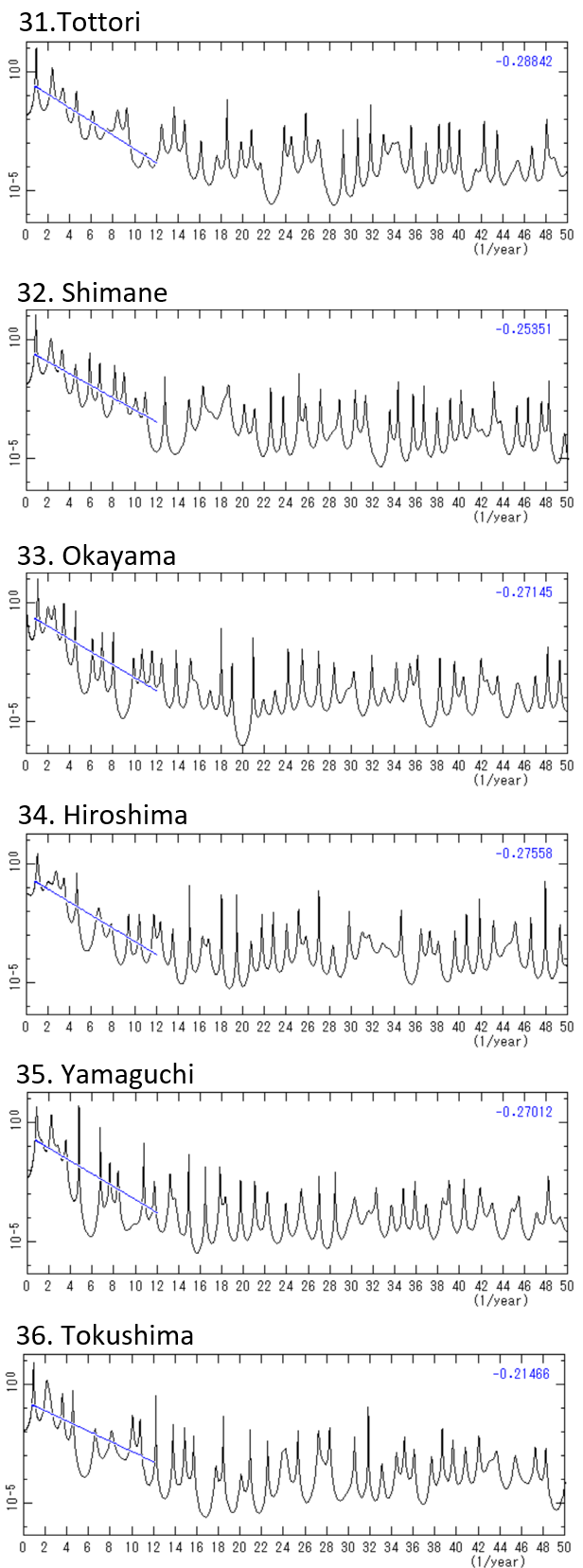 |
| --- | --- |

| 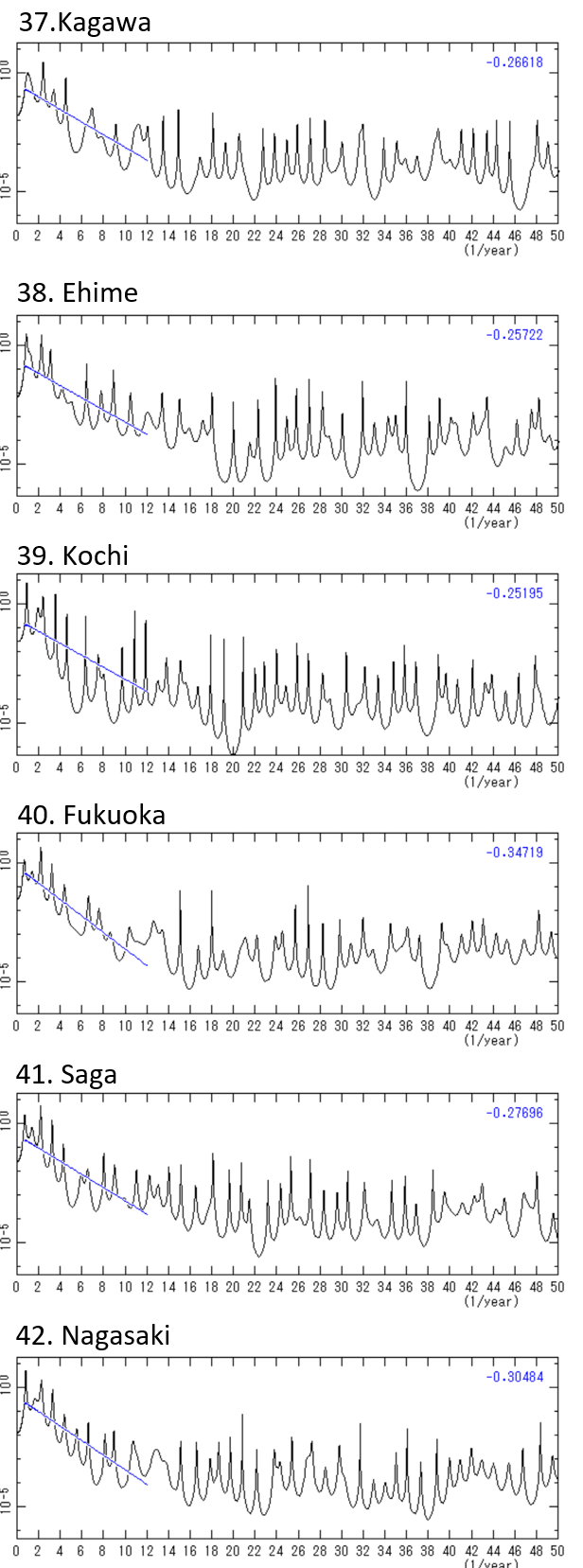 | 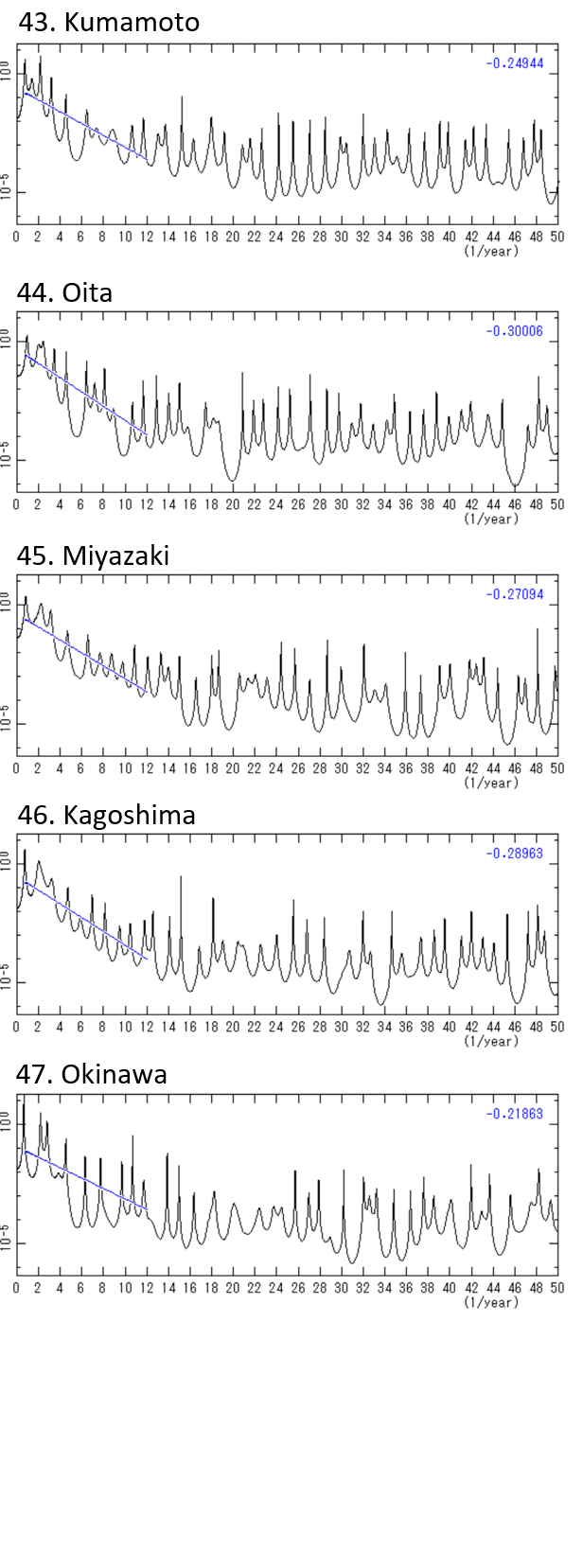 |
| --- | --- |

Supplement: S2 Fig — (DOCX) [file pone.0314233.s004.docx]
